# Assessment of the biochemical basis underlying the resistance against systemic amyloidosis

**DOI:** 10.1038/s41598-026-35297-9

**Published:** 2026-01-09

**Authors:** Tim Moderer, Adrian F. Schnell, Natalie J. Scheurmann, Matthias Schmidt, Christian Haupt, Nadine Schwierz, Marcus Fändrich

**Affiliations:** 1 Institute of Protein Biochemistry, Ulm University, 89081 Ulm, Germany; 2 Institute of Physics, University of Augsburg, 86159 Augsburg, Germany

**Keywords:** Inhibition, Prion, Protein aggregation, Systemic amyloidosis, Biochemistry, Biophysics, Structural biology

## Abstract

Amyloid resistance is the reduced or abrogated ability of an organism to develop a specific type of amyloid disease. For example, it was found that certain mouse strains show resistance against systemic AA amyloidosis, due to mutations in the amyloid fibril precursor protein serum amyloid A (SAA). In this research, we find that the SAA1.5 and 2.2, two SAA-isoforms that underlie amyloid resistance in mice are unable to adopt the structure of pathogenic AA amyloid fibrils under conditions, where the pathogenic protein variant SAA1.1 does so. Furthermore, molecular dynamics simulations show that the pathogenic fibril structure does not tolerate the sequential changes present in SAA1.5 and 2.2 and becomes distorted by the variant sequences. These data imply that SAA1.5 and SAA2.2 cause amyloid resistance due to a structural incompatibility of their sequences with the specific fold of pathogenic AA amyloid fibrils.

**Supplementary Information:**

The online version contains supplementary material available at 10.1038/s41598-026-35297-9.

## Introduction

Systemic AA amyloidosis is a globally occurring protein misfolding disorder caused by the formation of amyloid fibrils from serum amyloid A (SAA)1 protein^1–3^. SAA1 is an acute-phase protein whose serum levels can increase by a factor of 1,000 to 10,000 in response to a severe inflammatory stimulus^1,4,5^. Thus, chronic inflammatory conditions, such as rheumatoid arthritis, familial Mediterranean fever or tuberculosis are significant risk factors for the development of this disease in humans^4,6,7^. Systemic AA amyloidosis does not only occur in humans but also in animals (mammals and birds)^2,8^, where both captive as well as wild-living animals may be affected^2,8^. In particular murine AA amyloidosis has served for decades as a model for human diseases. It was used, for instance, to learn about the disease mechanism and to develop therapeutic strategies for systemic amyloidosis^9^. Moreover, certain mouse strains show amyloid resistance, that is, they have a reduced or abrogated ability to develop AA amyloidosis as a result of the expression of a variant SAA protein^10^.

There are several different SAA protein variants expressed in mice^11^, but amyloid formation has mainly been associated with SAA1 protein^12^. The protein may occur in different allelic variants with wildtype SAA, or SAA1.1, being the main amyloidogenic species. Other SAA variants may underlie amyloid resistance: SAA1.5 (sometimes referred to as SAA mm1 or SAA1B) was found to lead to amyloid resistance in *Mus* *musculus Czech*, SAA2.2 (sometimes referred to as SAA CE/J) in CE/J mice^10,13,14^. While several studies demonstrated that expression of these proteins is crucial for reducing the susceptibility to develop systemic AA amyloidosis^10,15,16^, there is uncertainty about the mechanism by which this happens, and inconsistent results and/or conclusions have been published by different groups^15–17^. For example, some studies claim amyloid resistance to arise from an intrinsic inability of SAA2.2 to form amyloid fibrils^16,18^. While these conclusions were based on observations that recombinant SAA2.2 constructs did not form amyloid fibrils in vitro or in cell culture^16,18^, other studies demonstrated the ability of SAA2.2 to form fibrils under in vitro conditions^19,20^.

**Fig. 1:**
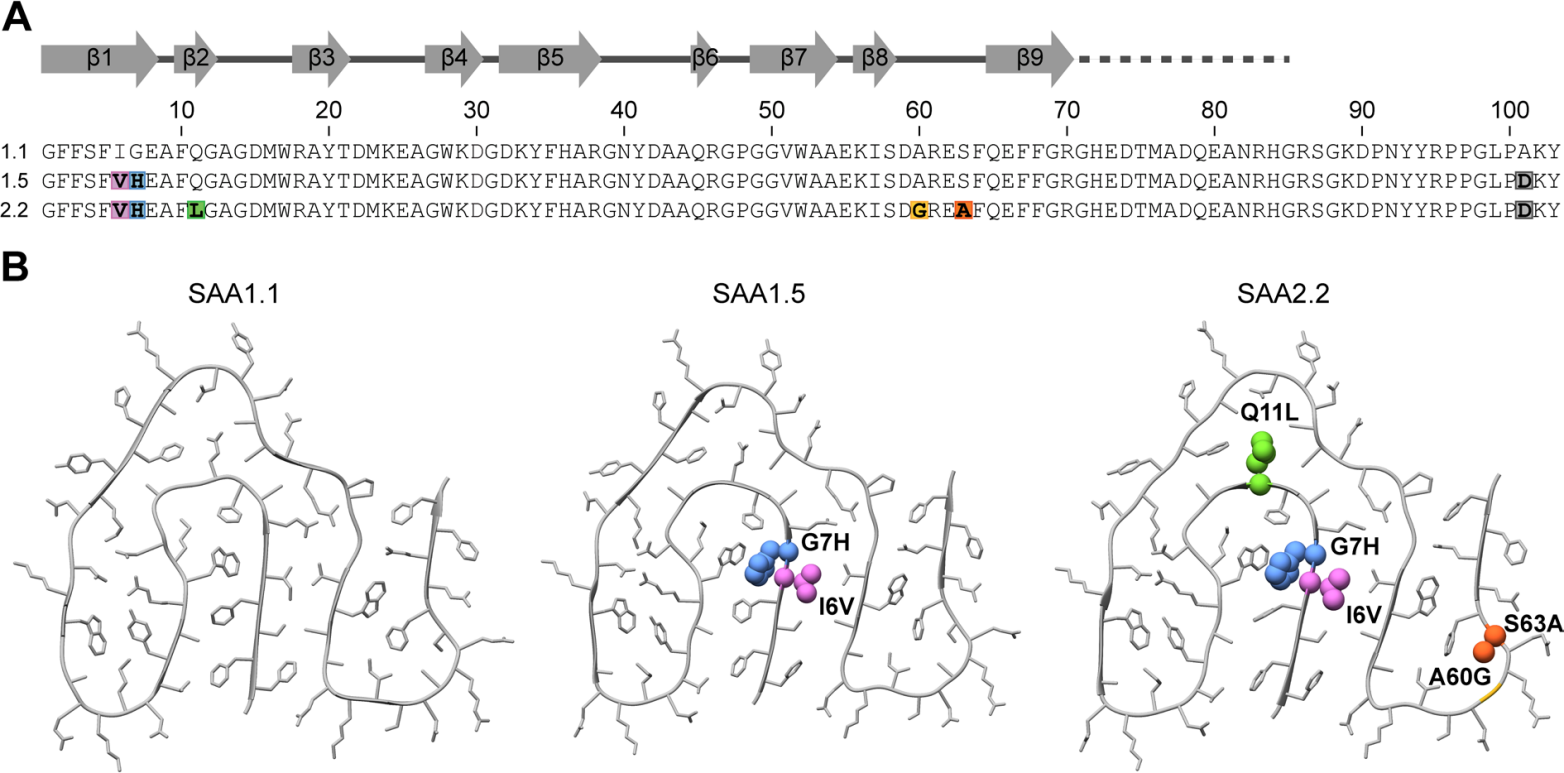
SAA isoforms and fibril homology models. (**A**) Sequence of SAA isoforms 1.1, 1.5 and 2.2 with the mutations highlighted in rainbow color. The continuous grey line indicates the residues in the fibril core. The dotted line indicates residues that are flexible in the fibril, but present in most fibrils. Additional C-terminal residues are missing in the fibril protein, probably due to proteolysis. (**B**) Ribbon diagrams of the ordered part of the experimentally verified cryo-EM structure of the AA fibril protein containing SAA1.1 (PDB entry 6DSO) and of the SAA1.5- and SAA2.2-based homology models. Mutations are highlighted in space-fill and in the same colors as in panel (**A**)

SAA1.5 and SAA2.2 differ from SAA1.1 by several mutational changes. SAA1.5 contains three changes (I6V, G7H and A101D), while there are six in SAA2.2 (I6V, G7H, Q11L, A60G, S63A and A101D, Fig. 1). This suggests that amyloid resistance could arise from those mutations that are common to both protein variants (I6V, G7H and A101D). A101D is unlikely contributing to this effect as the C-terminus of SAA1.1 is typically missing in murine AA amyloid fibrils^2,21^. The two remaining mutations I6V and G7H are, according to previous cryo-electron microscopy (cryo-EM) structures of murine AA amyloid fibrils^21,22^, located in the densely packed hydrophobic core of the fibril. Importantly, position 7 is only found in the core of fibrils extracted from amyloidotic tissue, which we refer to here as pathogenic AA amyloid fibrils, while murine SAA1.1 protein can form fibril morphologies in vitro that possess a different structure and that are not seen in fibril samples from amyloidotic tissue. In these (non-pathogenic) SAA fibrils, residue 7 is solvent-exposed^22^.

To test the hypothesis that the amino acid sequences of SAA1.5 and 2.2 are incompatible with the pathogenic fibril structure, we carried out in vitro aggregation studies with recombinant SAA1.1, SAA1.5 and SAA2.2 proteins in the presence and absence of fibrils, which were purified from an AA amyloidotic mouse. We find that SAA1.1, but not SAA1.5 and SAA2.2, is able to adopt the structure of pathogenic AA amyloid fibrils if seeded with these ex vivo fibrils. In addition, molecular dynamics (MD) simulations show that the mutational changes present in SAA1.5 and SAA2.2 are not tolerated by the fibril structure, resulting in pronounced structural deviations when these mutations are introduced.

## Results

### Recombinant SAA1.1 and 2.2 are able to form amyloid fibrils in vitro

**Fig. 2:**
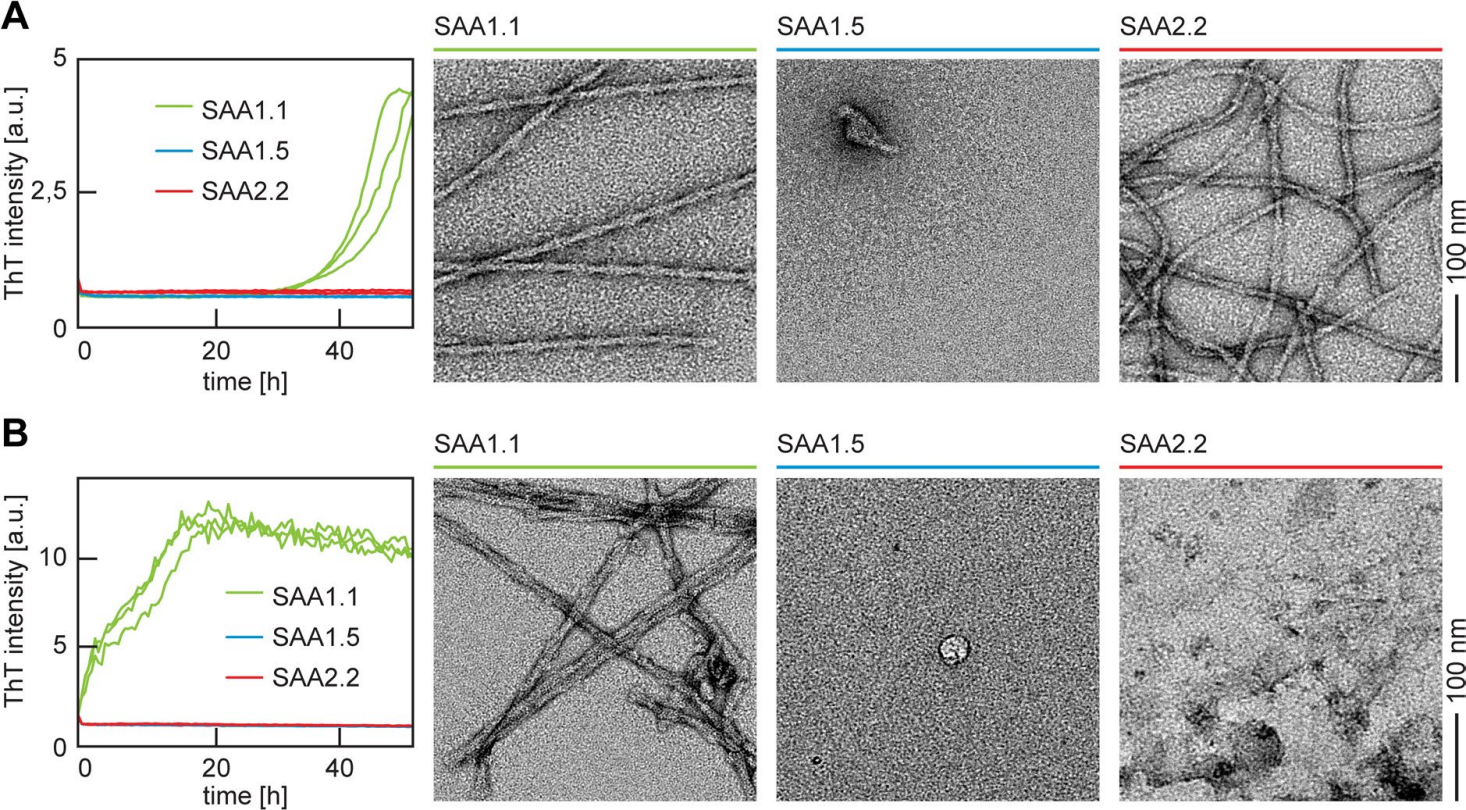
Fibril formation in vitro with or without ex vivo fibrils as seeds. (**A**) Left: time-resolved ThT fibrillation kinetics of recombinant SAA 1.1 (green), SAA1.5 (blue) and SAA2.2 (red) in the absence of seeds. Right: representative uranyl-acetate stained TEM images for the three types of samples. (**B**) Left: time-resolved ThT fibrillation kinetics of recombinant SAA 1.1 (green), SAA1.5 (blue) and SAA2.2 (red) in the presence of seeds. Right: representative uranyl acetate-stained TEM images for the three types of samples (for image of ex vivo SAA1.1 fibrils from splenic tissue see SI Fig. 2)

To determine the intrinsic fibrillation properties of SAA1.1, 1.5 and 2.2 proteins, we analyzed the residue-specific aggregation propensity of the three proteins with standard computational tools, such as Pasta 2.0^23^, aggrescan^24^, Waltz^25^, fold amyloid^26^ and AmylPred 2^27^. However, only small, if any, differences were observed in the general aggregation behavior of the three proteins. That is, the aggregation-prone segments were almost indistinguishable in the three proteins (SI Fig. 1). Much more pronounced differences were found if the three proteins were subjected to a biochemical analysis of their fibrillation behavior. This analysis used recombinantly expressed and purified SAA1.1, 1.5 and 2.2 proteins, which were incubated separately in 10 mM Tris buffer (pH 8.5). The samples contained also 20 µM of the amyloid binding, fluorescent dye Thioflavin T (ThT) so that amyloid formation could be monitored in real time over a period of 48 h (Fig. 2). Aliquots were withdrawn from each sample after incubation and analyzed with TEM. We found that reactions containing SAA1.1 gave rise to a notable increase in the ThT fluorescence that occurred with a lag time of 31.6 ± 0.6 h, indicating the formation of fibrils. Also transmission electron microscopy (TEM) revealed large quantities of long and straight filaments along with the presence of well resolved crossovers (Fig. 2A), consistent with previous observations^22^.

Incubation of SAA2.2 under the same set of conditions also led to the formation of fibrils as judged by TEM (Fig. 2A), but there was no discernible ThT increase (Fig. 2A). Incubation of SAA1.5 did not result in fibrils as judged by the ThT kinetic assay and the fluorescence intensity remained low throughout the experiment; and there was no evidence for fibril formation by TEM either (Fig. 2A). We conclude that SAA1.1 and 2.2 spontaneously nucleate the formation of amyloid fibrils in vitro, while SAA1.5 fails to do so, at least under the presently used set of experimental conditions.

### Ex vivo AA amyloid fibrils fail to proliferate their structure to SAA1.5 or SAA2.2 upon seeding

In a next step we repeated the kinetic experiment but added ex vivo AA amyloid fibrils, that were extracted from the spleen of an AA amyloidotic mouse (SI Fig. 2), as seeds to the freshly dissolved, recombinant proteins. Recombinant SAA1.1 protein showed potently accelerated fibril formation. There was no well-resolved lag phase visible by ThT (Fig. 2B), and TEM demonstrated the presence of fibrils after 48 h (Fig. 2B). Reactions containing SAA1.5 produced no substantial increase in their ThT fluorescence intensity upon seeding, and also TEM was not able to detect any fibrils (Fig. 2B). Samples containing SAA2.2 led to a uniformly low ThT signal if seeded with ex vivo AA amyloid fibrils and no fibrils could be observed by TEM in the majority of replicates. Occasionally, we found small quantities of fibrillar aggregates, possibly representing the seeds remaining until the end of the incubation period. However, out of the three proteins only SAA1.1 was reproducibly found to be able to form fibrils upon addition of ex vivo fibrils.

**Fig. 3:**
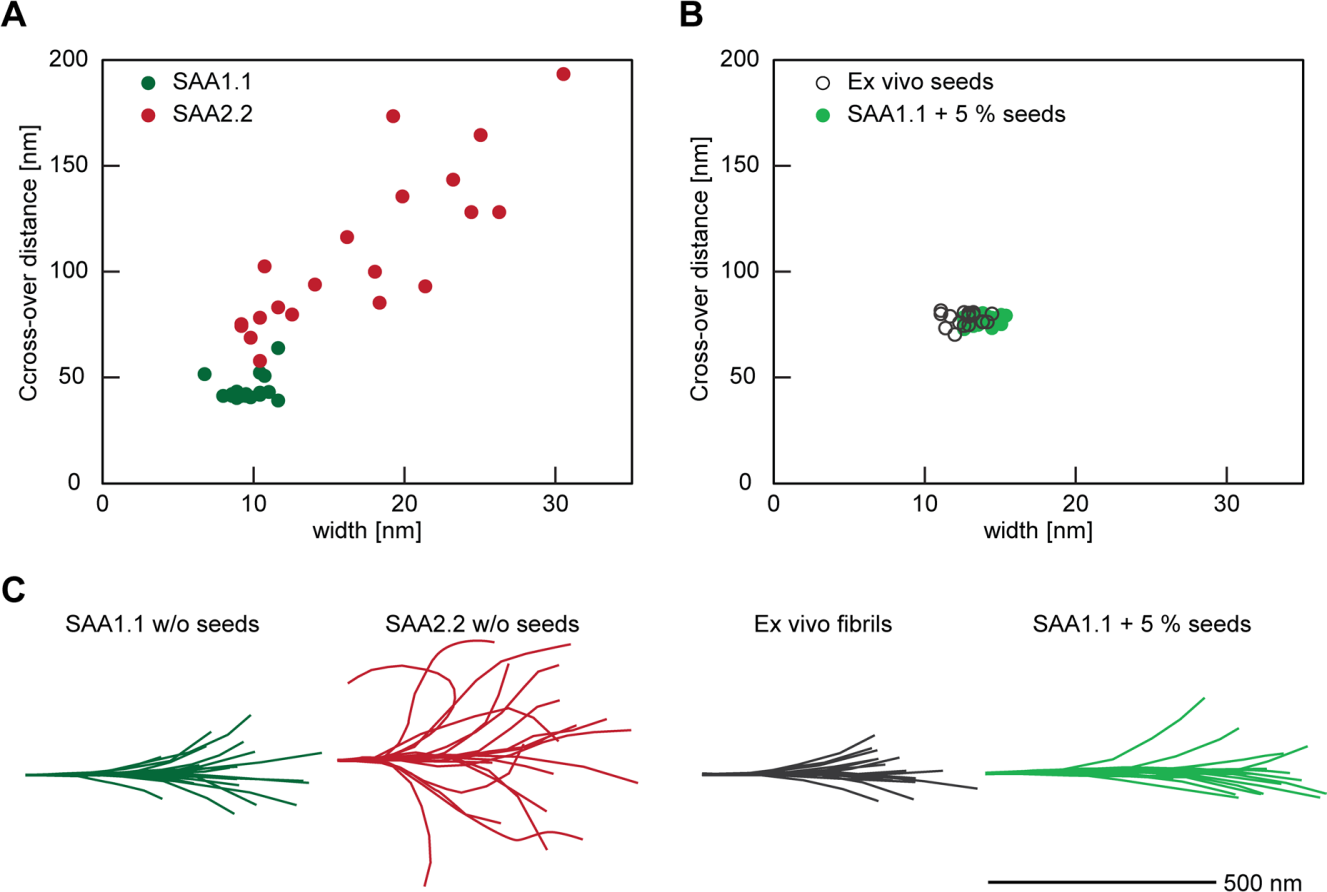
Morphological analysis based on cryo-EM. (**A**) Measurement of the crossover distance and fibril width based on the cryo-EM images of SAA1.1 (deep green) and SAA2.2 (red) fibrils formed in the absence of seeds (*n* = 20). Mean values for crossover distance and width are: 44.5 ± 5.7 nm and 9.5 ± 1.2 nm (SAA1.1) and 109.5 ± 38.7 nm and 17.0 ± 6.4 nm (SAA2.2). (**B**) Measurement of the crossover distance and fibril width of ex vivo fibrils (grey, hollow circles) and SAA1.1 fibrils (light green) formed in the presence of seeds (*n* = 20). Mean values for crossover distance and width are 78.0 ± 2.9 nm and 12.6 ± 0.9 nm (ex vivo) and 76.8 ± 2.3 nm and 12.8 ± 0.9 nm (SAA1.1 seeded). (**C**) Tracing of the fibril contour on the cryo-EM images of 20 single fibrils from unseeded SAA1.1 (light green), unseeded SAA2.2 (red), ex vivo (grey) and seeded SAA1.1 (light green).

To further investigate the structure of the formed fibrils we used cryo-EM (Fig. 3, SI Fig. 3). Unseeded SAA1.1 fibrils possessed a mean cross over distance of 44.5 ± 5.7 nm and a width of 9.5 ± 1.2 nm (*n* = 20, Fig. 3A, SI Fig. 3). These fibrils were also more tightly twisted and thinner than unseeded SAA2.2 fibrils, which showed a mean crossover distance of 109.5 ± 38.7 nm with a width of 17.0 ± 6.4 nm (*n* = 20, Fig. 3A, SI Fig. 3). SAA2.2 fibrils were more heterogeneous than SAA1.1 fibrils and showed substantially larger variations of the fibril widths and crossover distances (Fig. 3A). In addition, we noted that SAA1.1 and 2.2 fibrils present a significantly different bending behavior. Tracing of 20 representative fibrils on the recorded cryo-EM images revealed the unseeded SAA1.1 fibrils to be relatively straight, while a much more curvilinear and bent shape was found for unseeded SAA2.2 fibrils (Fig. 3C). Taken together with the different ThT fluorescence emission levels reported in Fig. 2A, we conclude that unseeded SAA2.2 fibrils are morphologically different and structurally more heterogeneous than unseeded SAA1.1 fibrils.

We then analyzed the seeded fibril samples. Seeding of recombinant SAA1.1 protein induced the formation of fibrils with a crossover distance of 76.8 ± 2.3 nm and a width of 12.8 ± 0.9 nm (*n* = 20, Fig. 3B, SI Fig. 3). Both values correspond, within error, to values measured with ex vivo AA amyloid fibrils that were used here as seeds (mean crossover distance of 78.0 ± 2.9 nm, average fibril width of 12.6 ± 0.9 nm, *n* = 20) and to literature values of these fibrils^21,28^. Moreover, seeded SAA1.1 fibrils showed the same bending behavior as the fibrils that were used as seeds (Fig. 3C).

Taken together with previously reported cryo-EM structures of ex vivo AA amyloid fibrils, and in vitro formed SAA1.1 fibrils after seeding with ex vivo fibrils^28^, we conclude that pathogenic AA amyloid fibrils are able to proliferate their typical fold to recombinant SAA1.1 protein. The susceptibility of SAA1.1 contrasts starkly to the properties of recombinant SAA1.5 or 2.2 proteins, which are both unable to adopt the ex vivo fibril structure and to be nucleated by the ex vivo fibrils (Fig. 2B). We conclude that these data provide strong support for the view that the amino acid sequences of SAA1.5 and 2.2 are incompatible with the fold of pathogenic AA amyloid fibrils.

### MD simulations show that SAA1.5 and 2.2 disrupt the fibril core

**Fig. 4:**
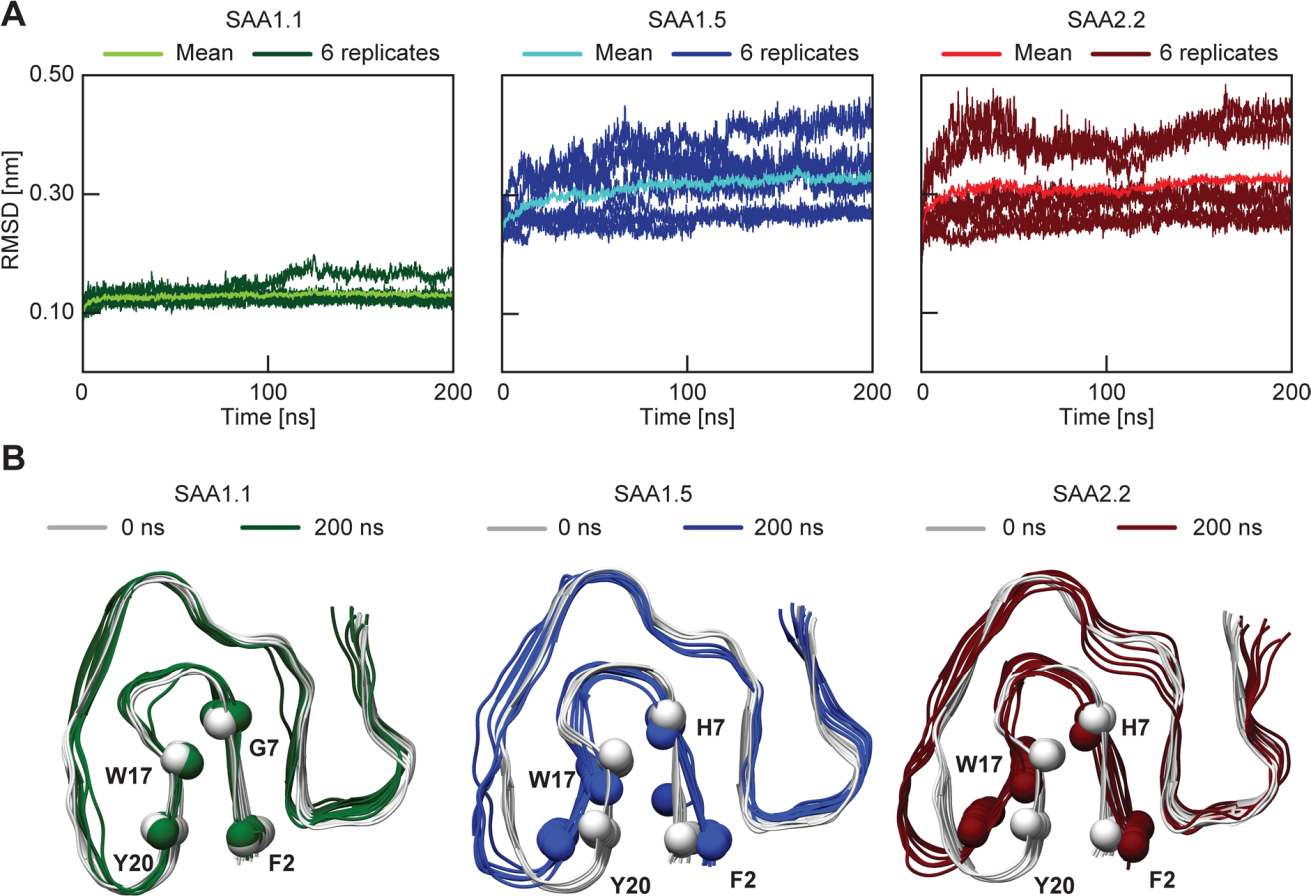
Comparison between the SAA1.1 fibril structure and homology models based on SAA1.5 and 2.2. (**A**) RMSD trajectories of six-layer fibril protein stacks of the SAA1.1 fibril (green) and SAA1.5- (blue) and SAA2.2-based homology models (red) as obtained from the MD simulation. Dark color: six RMSD trajectories that arise from three simulations per fibril variant, each containing two fibril protein stacks. Light color: mean RMSD values over the period of 200 ns. (**B**) Overlay of the starting point of the simulation (0 ns, grey) and the final models at the endpoint of the simulation (200 ns) to illustrate the outward movement of the β-arch. One stack per fibril variant is shown: SAA1.1, green; SAA1.5, blue; SAA2.2, red. The Cα atoms selected for distance measurements (see SI Fig. 3) are highlighted with spheres.

To further test this conjecture, we conducted all-atom MD simulations of the experimentally determined SAA1.1-derived AA amyloid fibril (PDB entry 6DSO) and of two homology models of this structure that were based on the sequences of SAA1.5 and 2.2 (Figs. 1B and 4). Analysis of the Cα atom root mean square deviation (RMSD) between the simulated fibrils after 100 ns and the conformation at the beginning of the simulations revealed only small deviations (~ 0.13 nm) for the cryo-EM structure, which was derived from SAA1.1 protein (Fig. 4A). Considerably larger RMSD values (> 0.3 nm) were obtained when analyzing the MD simulations of the SAA1.5- and SAA2.2-based homology models (Fig. 4A). These data imply that the pathogenic, SAA1.1-based fibril retains its structure over the course of the MD simulation, while the SAA1.5 and 2.2-based homology models undergo conformational changes.

An overlay of stacks of the fibril protein before and after simulation reveals only small differences in case of SAA1.1-derived structures, while several SAA1.5 and 2.2-based simulations show a substantial outward movement of the β-arch, that is formed by residues D15 to D32 (Fig. 4B). This outward movement leads to a dissociation of the arch, from the fibril protein N-terminus on residues G1 to E8 (Fig. 4B). Measuring the Cα-Cα atom distances between residues 7 (G or H) and W17 confirmed this observation and showed larger distances in the SAA1.5 and SAA2.2-based models than in the SAA1.1-derived fibril (SI Fig. 4). A similar trend is seen for the Cα-Cα distances between residues F2 and Y20 (SI Fig. 3). Associated with this movement is a rearrangement of the side chain rotamers of W17 and H7 in the SAA1.5- and 2.2- based models (SI Fig. 4). No such rotamer rearrangement is seen for the SAA1.1-based simulations (SI Fig. 4). In summary, both SAA1.5 and SAA2.2-based models show structural distortions, while the SAA1.1-derived fibrils remain largely unchanged and closely resemble the ex vivo structure throughout the simulations.

## Discussion

In this study we investigated whether or not recombinantly expressed SAA1.1, 1.5 and 2.2 proteins are able to form fibrils in vitro that resemble the fibril structure of pathogenic AA amyloid fibrils. We find that SAA2.2 is able to form fibrils in vitro (Fig. 2), similar to previous observations from the Colón group^17,19,20^, although the structure of SAA2.2 fibrils is different from the morphology adopted by SAA1.1 (Fig. 2, SI Fig. 3). In the case of SAA1.5, we found no formation of fibrils under any of the experimental conditions tested here (Fig. 2, SI Fig. 3). The formation of fibrils from SAA2.2 protein in vitro rules out that the amyloid resistance arises simply from an intrinsic inability of the protein to form amyloid fibrils. Hence, a more refined explanation must be provided in order to rationalize the phenomenon of amyloid resistance.

The most notable difference of SAA2.2 and 1.5 compared to SAA1.1 is that the two mutant proteins are unable to proliferate the structure of ex vivo amyloid fibrils in a seeding experiment (Fig. 2B, SI Fig. 2). This inability is in particular surprising for SAA2.2 as the protein is able to fibrillate in the absence of seeds (Fig. 2A), suggesting that the ex vivo fibrils are not only unable to serve as a template for fibril seeding, but rather exert an additional inhibitory effect that suppresses the de novo nucleation of SAA2.2. While the molecular basis of the inhibitory effect remains to be established - it may actually arise from tissue-derived secondary components that were not removed in the course of the fibril purification - it is clear that ex vivo fibrils are not able to seed SAA1.5 and 2.2.

Our data provides evidence that the sequences of SAA1.5 and 2.2 are incompatible with the specific morphology of pathogenic AA amyloid fibrils. That is, amyloidosis is prevented in these animals as the variant SAA proteins are not able to form the specific fibril morphology that is required for pathogenicity. Indeed, previous research established that amyloid fibril precursor proteins, such as murine SAA1.1, spontaneously form fibril morphologies in vitro that do not necessarily match the fibrils seen in a patient or diseased animal^22,29,30^. Therefore, out of the spectrum of possible fibril morphologies that can be found by a given protein only some lead to amyloidosis. If these fibril structures cannot be adopted by a mutant amino acid sequence, expression of this protein causes amyloid resistance in the affected organism.

This explanation is supported here by two other observations. First, there is no major difference in the intrinsic aggregation propensity of the three proteins as revealed by classical sequence analysis tools (SI Fig. 1). These tools calculate the general tendency to form aggregated β-sheet conformation within a sequence segment but they do not calculate the propensity to adopt a specific fibril morphology^31–33^. Second, the incompatibility of SAA1.5 and SAA2.2 with the pathogenic fibril structure is also found in MD simulations which show rearrangements of the fibril models if these are constructed from the variant proteins (Fig. 4A).

Amyloid resistance upon mutation has been reported for a variety of amyloid diseases, such as Creutzfeldt-Jakob^34^, Kuru^35^, Alzheimer’s disease^36,37^ and ApoA-II amyloidosis^38^, and there are cases of this phenomenon in both humans and animals. Not all of these examples may follow the same mechanism that is revealed here for murine systemic AA amyloidosis. For example, some mutations seem to act by provoking an altered cellular processing of the amyloid fibril precursor protein^36,37^. In case of murine systemic ApoA-II amyloidosis, however, there is evidence for a similar mechanism of amyloid resistance as we describe here. This amyloidosis is seen in mice expressing the apolipoprotein AII (ApoA-II) variant ApoA-IIC^39^, whereas mice expressing ApoA-IIF show resistance against the disease^40^. Moreover, it was found that the amino acid sequence of the amyloid-resistant animal (ApoA-IIF) is incompatible with the pathogenic fibril structure^38^.

Amyloid resistance due to SAA1.5, SAA2.2 or ApoA-IIF may be a dominant phenomenon; that is, the resistance may be seen in animals that express the amyloid-resistant protein together with an amyloid-sensitive one^10,40,41^. In case of SAA2.2 however, conflicting literature exists on this issue as some researchers claim a dominant inhibitory effect when SAA1.1 and 2.2 are co-expressed in mice heterozygous for both SAA isoforms^41^, while others report the lack of this– dominant effect in mice co-expressing SAA1.1 and 2.2^18^ or when SAA2.2 is introduced via a recombinant adenoviral expression system into mice homozygous for SAA1.1. Although the reasons for this discrepancy remain to be established, it is possible that the heterozygous expression of SAA2.2 delays rather than prevents fibril growth, as an inhibition of fibril formation was observed on day three after amyloidosis induction^41^, but not after five days^16^, or ~ five weeks^18^. A dominant effect of the variant protein could be scientifically interesting as it implies that the introduction of an amyloid-resistant protein, or fragments thereof, inhibit amyloid formation. Indeed, injection of an 18-residue peptide fragment from an amyloid-resistant variant of ApoA-II protein into mice, that express the amyloid-susceptible protein variant ApoA-IIC, was found to antagonize amyloid formation in the recipient animal^40^. Amyloid resistance may thus therefore be exploited as a therapeutic principle, at least in some types of amyloid diseases.

## Methods

### Prediction of aggregation-prone regions

To identify the aggregation prone regions of SAA1.1, 1.5 and 2.2 we used the five different computational programs PASTA 2.0^23^, Aggrescan^24^, Waltz^25^, FoldAmyloid^26^ and AmylPred 2^27^ using the following settings: PASTA 2.0, thresholds with 90% specificity and an energy cutoff value of − 2.8 (maximum); Aggrescan, residues with an a4v value of greater than − 0.02 were counted as hits; WALTZ, residues with a score greater than 0.00; FoldAmyloid, 5 or more consecutive residues with a score greater than 21.4; AmylPred 2, the consensus method was used to assign a residue as a hit.

### Source and preparation of recombinant murine SAA protein

Wild-type SAA1.1 protein as well as SAA1.5 and SAA2.2 were recombinantly expressed as fusion proteins in *Escherichia coli* RV308 cells and purified via column chromatography, as described previously^42^. The purified protein was stored in a lyophilized form at − 80 °C and dissolved in water at an initial concentration of 1 mg/mL immediately prior to the experiment.

### Generation of AA amyloidotic mice

Amyloidosis was induced in Naval Medical Research Institute (NMRI) mice homozygous for SAA1.1. 10 to 14 weeks old female NMRI mice weighing approximately 35 g were obtained from Charles River Laboratories. On day 0 of the experiment each mouse received a single injection of 0.1 mL water, containing 10 µg murine AA amyloid fibrils into the lateral tail vein. Immediately afterwards, the animals received a subcutaneous injection of 0.2 mL freshly prepared 1% (w/v) solution of AgNO_3_ in distilled water. The AgNO_3_ injection was repeated after 7 and 14 days. Animals were anesthetized and euthanized with CO_2_ on day 16 and spleens were removed subsequently. All animal experiments were approved by the Regierungspräsidium Tübingen (TVA #1628) and methods were conducted in accordance with their guidelines and regulations as well as the ARRIVE guidelines.

### Preparation of ex vivo fibrils

Ex vivo fibrils were extracted from AA amyloidotic mice as described previously^43^. In brief, 250 mg of splenic tissue material were washed ten times with 1 mL tris(hydroxymethyl)-aminomethane (Tris) calcium buffer (20 mM Tris, 138 mM NaCl, 2 mM CaCl_2_, pH 8.0). In between each washing step, the samples were centrifuged at 3,100 × *g* for 5 min at 4 °C. The final pellet was resuspended in 1 mL freshly prepared collagenase/protease inhibitor solution [one protease inhibitor ethylenediamine-tetra acetic acid (EDTA)-free tablet (cOmplete™, Roche) in 7 mL Tris Calcium Buffer, 5 mg/mL crude collagenase from *Clostridium histolyticum* (Sigma)]. After overnight incubation at 37 °C (horizontal shaking at 750 rpm) the tissue material was centrifuged at 3,100 × *g* for 30 min at 4 °C. The pellet was resuspended in 500 µL Tris EDTA buffer (20 mM Tris, 140 mM NaCl, 10 mM EDTA, pH 8.0), homogenized and centrifuged for 5 min at 3100 × *g* at 4 °C. This step was repeated three times. Afterwards, the tissue pellet was resuspended in 500 µL ice cold water and centrifuged for 5 min at 3,100 × *g* at 4 °C. The fibril containing supernatant was stored at 4 °C. This step was repeated four times. The fibril concentration of the supernatant was obtained by densitometric analysis of a denatured gel electrophoresis gel (Bis-Tris 4–12% gradient gel, NuPAGE™) with recombinant SAA1.1 as concentration standard.

### In-vitro aggregation assay

20 µL of the 1 mg/mL SAA stock solutions in water were pipetted into each well of a black wall 96-well plate (Greiner Bio-One) and mixed with 80 µL (unseeded reaction) or 70 µL (if seeds were added) from a kinetic master mix (containing Tris buffer, pH 8.5, ThT and water) to reach a final concentration of 10 mM Tris pH 8.5, 20 µM ThT. For the seeding experiments 5% (w/w) seeds (10 µL with a concentration of 0.1 mg/mL), obtained from mouse tissue (see above) were added before the incubation started. After mixing of the samples in the wells by pipetting up and down, the plate was covered with a sealing film (Rotilabo^®^) and incubated at 37 °C in a FLUOstar Omega plate reader (BMG Labtech) using the following settings: bottom optic, excitation filter: 450 nm, emission filter: 490 nm, orbital averaging: 4 mm, 20 flashes per well and cycle, total cycle time: 1,800 s.

### Negative-staining transmission electron microscopy (TEM)

After completion of the in vitro aggregation assays (see above) 5 µL aliquots were taken from the 96-well plate and transferred onto formvar and carbon coated 200 mesh copper grids (Electron Microscopy Sciences). The samples were incubated at room temperature for 30 s, remaining solution was blotted away using filter paper (Whatman), followed by three washing steps in a water droplet and three staining steps in a 50 µL droplet of 2% (w/v) uranyl acetate. Using filter paper, the excess fluid was removed after each step. As a last step, the samples were air-dried for 10 min and inquired at 120 kV in a JEM-1400 transmission electron microscope (JEOL).

### Cryo-EM

Aliquots of the fibril solution (3.0 µl) were applied to glow-discharged (PELCO easiGlow™, Ted Pella, Inc.) 1.2/1.3 holey carbon coated grids (Protochips). After 30 s incubation at a humidity of 95% the fibril solution was blotted for 8 s from the back side and plunge frozen into liquid ethane using EM GP2 plunge freezer (Leica Microsystems GmbH). Images of the prepared grids were taken on a JEM-2100 TEM (JEOL) with a TemCam-F416 (TVIPS).

### Tracing of the fibrils on the micrographs

The respective cryo-TEM images were opened with Adobe^®^ illustrator and 20 fibrils were manually traced using the ‘line tool’ for ~ 500 nm for each condition. The resulting fibril traces were aligned to visualize the structural difference between the different proteins/conditions.

### Molecular dynamics simulation

We used all-atom MD simulations to characterize the conformational dynamics of amyloid fibrils composed of three different sequences (SAA1.1, SAA1.5, and SAA2.2). Following the cryo-EM structure of murine AA amyloid fibril (PDB: 6DSO), all fibrils were simulated as two stacks, each with six layers. SAA1.5 and 2.2-based fibrils were modeled using LocalColabFold^44,45^, leveraging one half-layer (69 residues) from the 6DSO structure as a template. We then constructed the complete 12-mer fibril structure by aligning the monomers predicted by LocalColabFold with the 6DSO 12-mer structure; i.e. two fibril protein stacks each containing six layers. Protonation states of ionizable residues were assigned with PROPKA v3.5.1^46,47^ as implemented in the APBS webserver (https://server.poissonboltzmann.org/) at pH 7.4, using the AMBER forcefield option.

We performed three independent 200 ns MD simulations for each fibril variant. Each fibril was placed in a tetragonal box, ensuring a minimum distance of 2.5 nm between protein and the box edge. All systems were solvated with optimal point water (OPC) water^48^ and 150 mM NaCl. Ion parameters from Mamatkulov & Schwierz^49^, initially optimized for transferable intermolecular potential with three points (TIP3P) water were used only after we verified that the parameters also reproduce experimental solvation free energies and activity derivatives when combined with OPC water. The protein force field parameters were taken from the AMBER ff19SB force field^50^, protein topology files were created using AmberTools23^51^.

MD simulations were performed with GROMACS 2024.2^52^. The initial structures were energy–minimized using the Conjugate Gradient and Steepest Descent algorithms preceding 2 ns with constant number of particles, volume and temperature (NVT) and constant number of particles, pressure and temperature (NPT) equilibration phases. During these equilibration runs, position restraints (1,000 kJ/mol*nm^2^) were applied to protein heavy atoms. Subsequently all position restraints were released, and production runs were carried out in the NPT ensemble at 310.15 K – matching the physiologically relevant temperature. The temperature was maintained with the velocity-rescaling thermostat^53^ using a time constant of 1 ps, pressure was controlled by means of the Parrinello-Rahman barostat^54^ with a coupling constant of 5.0 ps and a reference pressure of 1 bar. Periodic Boundary Conditions were applied in all directions; long range electrostatics were calculated with the Particle-mesh Ewald method^55,56^. All bonds involving hydrogen were constraint with the LINCS algorithm^57^, permitting the use of a 2 fs integration time step.

RMSD calculations were done using MDAnalysis v2.8.0^58–61^. Each stack of the simulated fibrils was analyzed separately after alignment to the experimental 6DSO structure as a reference. Only Cα atoms were considered.

### Cα-Cα distances measurements

For the measurement of the Cα-Cα distances the models of the initial structures as well as of the endpoints of the simulation were analyzed with the software university of California San Francisco (UCSF) Chimera. Cα-Cα distances between residues G7 (or H7) and W17 as well as between F2 and Y20 of the four central layers of both stacks of each model were determined by selecting both Cα atoms and measuring the distance using the structure analysis tool ‘distances’. This led to a total of eight single values for the Cα-Cα distances for each model and each simulation run. Statistical significance was determined using a paired t-test and *p* < 0.05.

## Supplementary Information

Below is the link to the electronic supplementary material.

Supplementary Material 1

## Data Availability

The datasets used and/or analyzed during the current study are available from the authors on reasonable request.
