# Supplementary material for: Assessment of the biochemical basis underlying the resistance against systemic amyloidosis
Source: Sci Rep. 2026 Jan 9;16:1313. doi: 10.1038/s41598-026-35297-9 (PMC12796397; doi:10.1038/s41598-026-35297-9)
Supplement: Supplementary file 1 — Supplementary Material 1 [file 41598_2026_35297_MOESM1_ESM.pdf]

Supplementary Information

Assessment of the biochemical basis underlying the resistance against systemic amyloidosis

Tim Moderer[1, *], Adrian F. Schnell[2], Natalie J. Scheurmann[1], Matthias Schmidt[1], Christian Haupt[1], Nadine Schwierz[2], and Marcus Fändrich[1]

[1]*Institute of Protein Biochemistry, Ulm University, 89081 Ulm, Germany*
[2]*Institute of Physics, University of Augsburg, 86159 Augsburg, Germany*
[*]*Corresponding author: tim.moderer@uni-ulm.de*

Supplementary Figures

Supplementary Figure S1

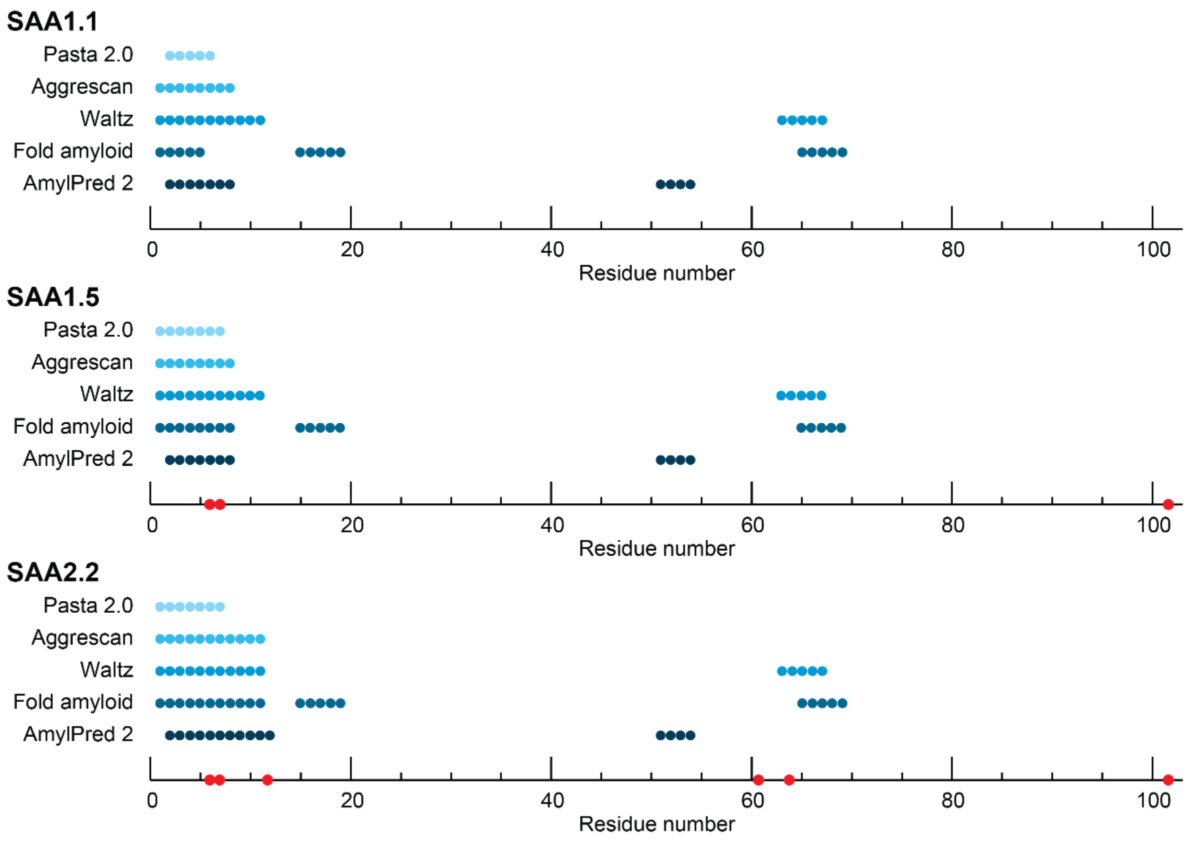

Supplementary Figure S1.

Aggregation propensity per residue for SAA1.1, 1.5 and 2.2 proteins.

Aggregation propensity of SAA1.1, 1.5 and 2.2 proteins calculated with the programs Pasta 2.0[23], aggrescan[24], Waltz[25], FoldAmyloid[26] and AmylPred[27] as indicated in the figure. Mutant positions are indicated by red dots.

Supplementary Figure S2

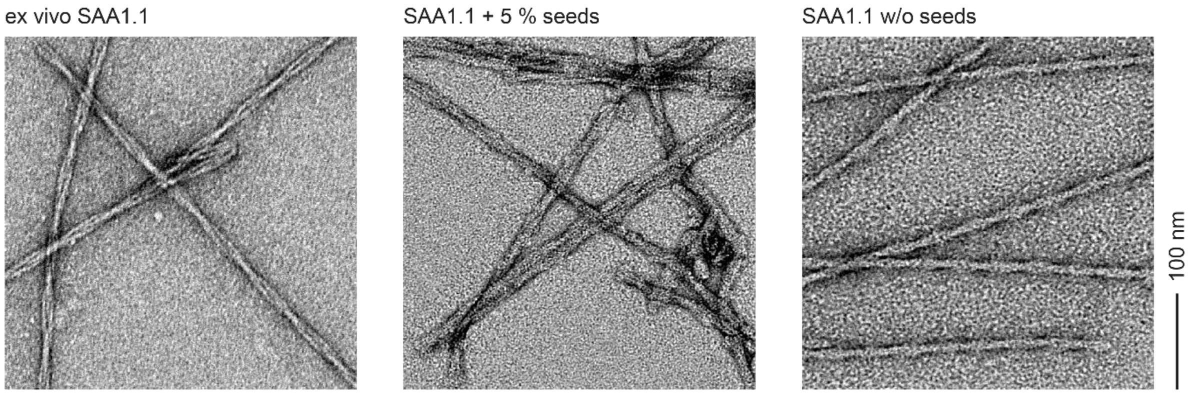

Supplementary Figure S2.

Uranyl-acetate stained TEM images of fibrils from SAA1.1.

Left: SAA1.1 fibrils extracted from murine splenic tissue. Middle: SAA1.1 Fibrils formed in vitro in the presence of 5 % ex vivo fibrils as seeds. Right: SAA1.1 fibrils formed in vitro in the absence of seeds.

Supplementary Figure S3

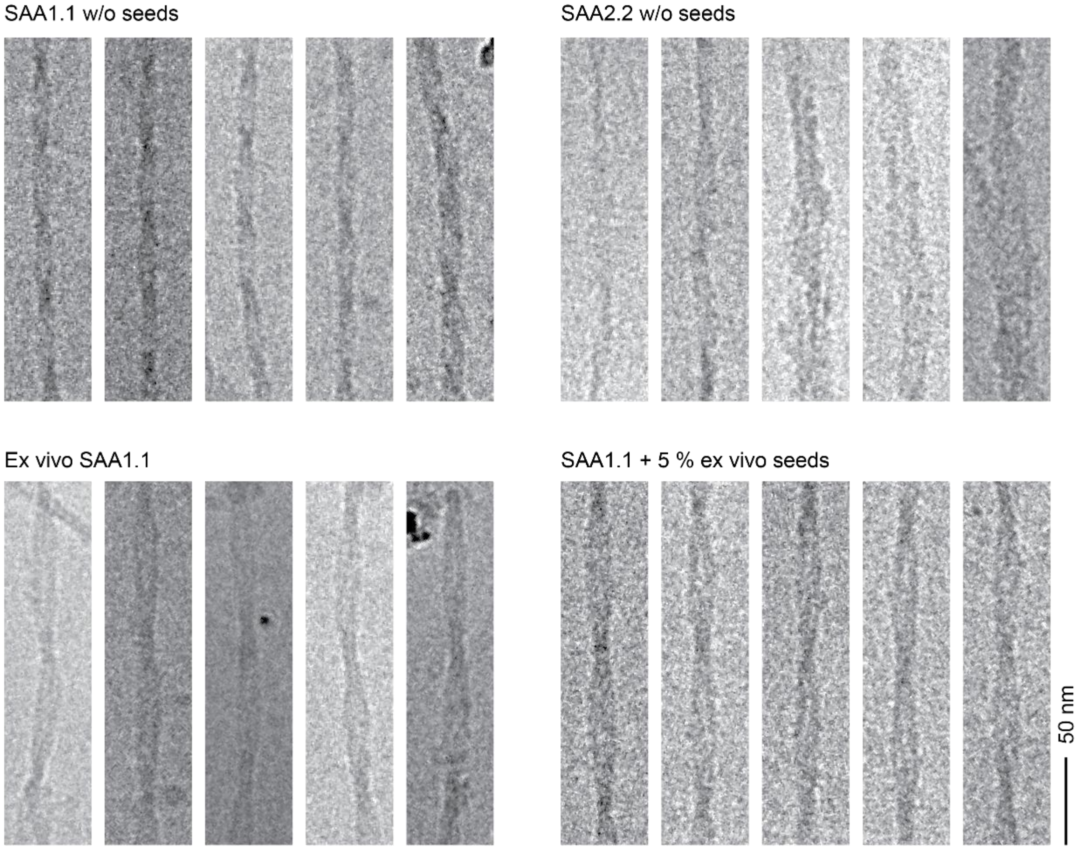

Supplementary Figure S3.

Cryo-EM images of the analyzed fibrils.

Five representative 200 kV cryo-EM images of SAA1.1 and 2.2 fibrils formed without seeds as well as SAA1.1 fibrils purified from diseased mice and SAA1.1 fibrils formed in the presence of 5 % (w/w) seeds.

Supplementary Figure S4

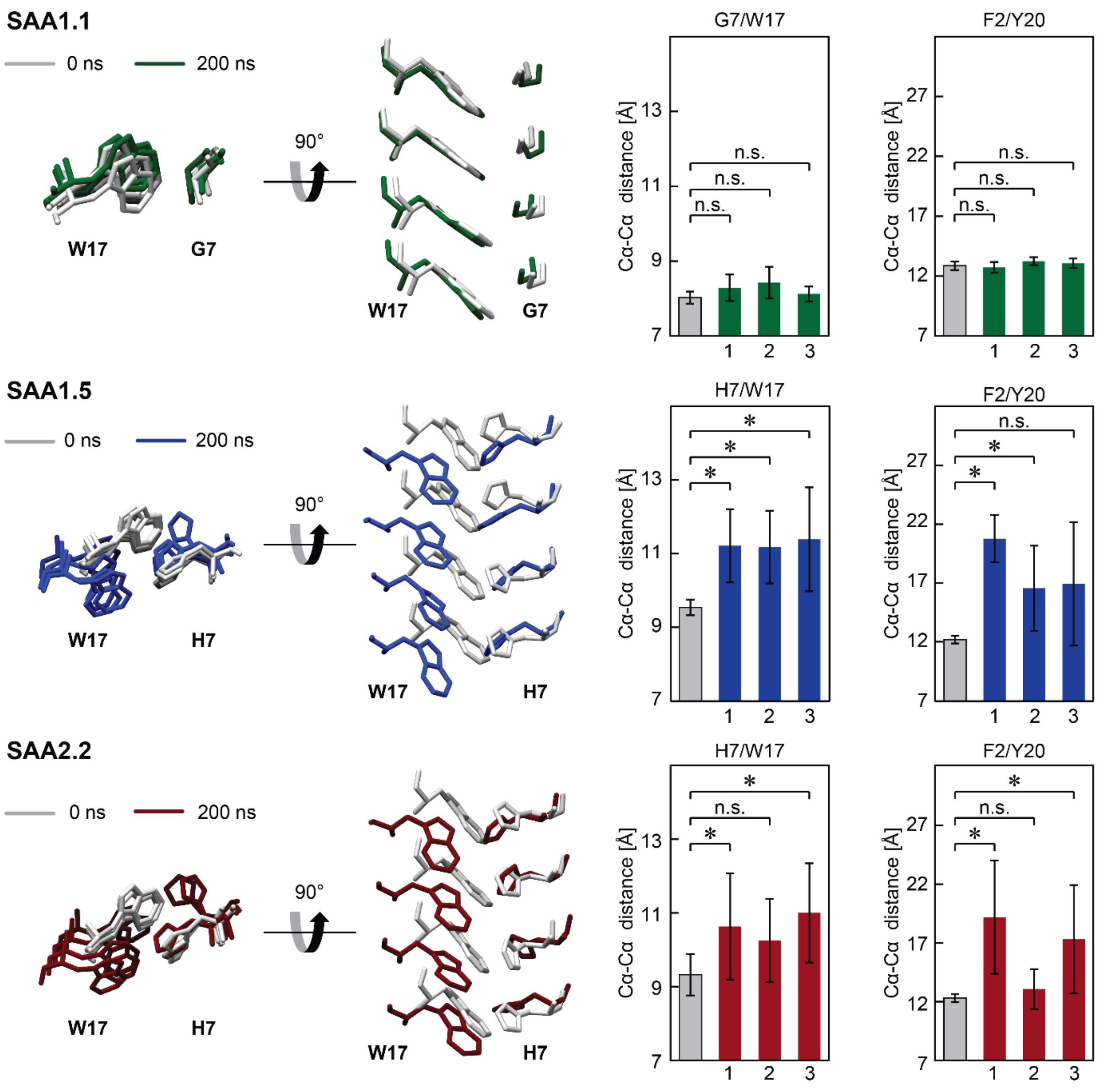

Supplementary Figure S4.

Structural rearrangements observed in the MD simulations.

Left: Top and side view of residues G7 (or H7) and W17 before (grey) and at the end of the simulation (green, SAA1.1; blue, SAA1.5; red, SAA2.2). Only the four central layers of one fibril stack are shown. Right: The histograms show the Cα-Cα atom distances between residues G7 (H7) and W17 as well as between F2 and Y20 of the four central layers of the three fibril

variants at the beginning (grey bars) and at the end of the simulation (filled bars), error bars indicate the standard deviation. Significant differences ($p<0.05$) between the Cα-Cα distances before ($n=8$, grey) and after three simulations ($n=8$, green, blue, red) are indicated by asterisks (*), the other differences are non-significant (n.s.). Statistical analysis was performed using a paired t test.
